# Assessment of the safety of long-acting β_2_-agonists in routine asthma care: the ASTRO-LAB protocol

**DOI:** 10.1038/npjpcrm.2015.40

**Published:** 2015-06-18

**Authors:** Eric Van Ganse, Nathalie Texier, Alexandra L Dima, Laurent Laforest, Montserrat Ferrer, Gimena Hernandez, Stéphane Schuck, Sandrine Herbage, Delphine Vial, Marijn de Bruin, and the ASTRO-LAB group

**Affiliations:** 1 Lyon Pharmaco-Epidemiology Unit- UMR 5558 CNRS-Claude Bernard Lyon 1 University, Lyon, France; 2 Respiratory Medicine, Croix-Rousse University Hospital, Lyon, France; 3 Kappa Santé, Paris, France; 4 Amsterdam School of Communication Research ASCoR, University of Amsterdam, Amsterdam, The Netherlands; 5 IMIM—Hospital del Mar Medical Research Institute, Barcelona, Spain; 6 Universitat Autónoma de Barcelona, Barcelona, Spain; 7 Cegedim Strategic Data Medical Research Limited, London, UK; 8 Aberdeen Health Psychology Group, University of Aberdeen, Aberdeen, UK

## Background

The safety of long-acting β_2_-agonists (LABAs) remains controversial in asthma, particularly in children, which led regulators to contraindicate LABAs as a single agent in asthma treatment.^1^ Current evidence regarding the safety of LABAs in combination with inhaled corticosteroids (ICs), based on meta-analyses of randomised clinical trials (RCTs), is less consistent. Recently updated meta-analyses for formoterol and salmeterol failed to reassure on safety.^2,3^ Despite the absence of evidence of serious risk with LABAs associated with ICs, the precision of results was low owing to infrequent outcomes. Furthermore, RCTs include highly selected populations, and they are not properly designed to assess infrequent and/or long-term adverse events in actual conditions of drug use.^4^

Evidence is also limited in the observational context. A recent systematic review assessing the risk of LABAs associated with ICs, compared with ICs alone, did not indicate any increased risk for emergency visits or hospital admissions.^5^ However, no reliable conclusions could be drawn neither in children, nor on potential differences between LABAs associated with ICs, in fixed-dose combinations, and in two separate canisters, due to the lack of published data for these specific issues. This review also highlighted the scarcity of prospective studies and the lack of data on drug adherence. Most of the observational studies were based on claims databases, providing only a partial assessment of drug exposure. There is an evidence gap, as detailed and valid exposure data are needed. For instance, irregular use of ICs in persistent asthma is a well-known source of exacerbations.^6^ Thus, there is a need to explore potential risks associated with LABAs in real life, with more extensive assessments of patterns of use, including ICs concomitant therapy, asthma control and exacerbations over time.

## Aims

The ASTRO-LAB project aims to provide new evidence about the safety of LABAs in children and adults in routine clinical care. Its main objective is to investigate with prospective data whether asthma patients receiving LABAs are at a higher risk of severe asthma exacerbations (SAEx), taking into account baseline differences in severity. Potential variations of respective drug exposures to LABAs and ICs over time, using complementary data sources will also be investigated. In addition, it will be verified whether differential adherence to LABAs and to ICs is a possible mechanism of increased risk of SAEx and other asthma outcomes in patients using these drugs in two separate canisters. A key question that ASTRO-LAB aims to explore is whether the potential LABA-associated risk can be explained by suboptimal adherence to ICs.

## Methods

ASTRO-LAB is a 24-month prospective observational study in asthma, conducted in France and in the United Kingdom (UK).

### Participants

**Figure 1:**
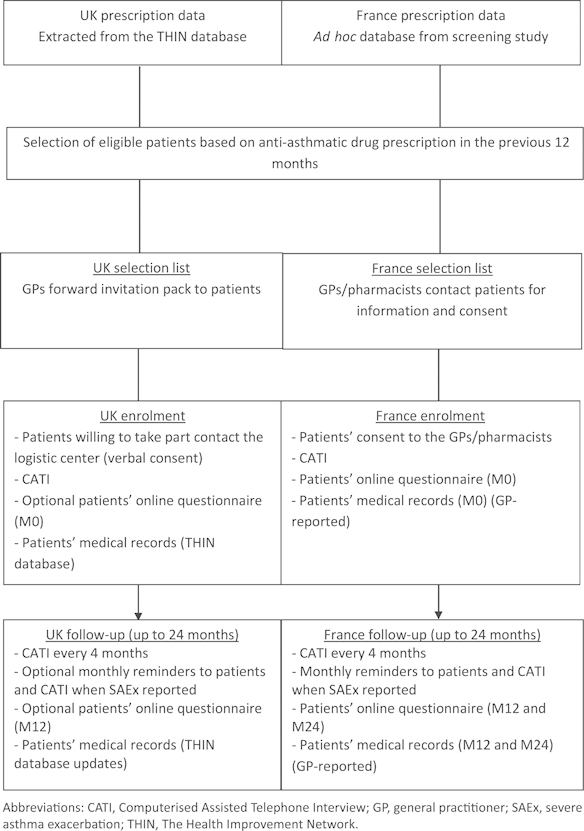
Study flowchart for patient enrolment and follow-up.

ASTRO-LAB will include persistent asthma patients treated in primary care, equally distributed between children (6–17 years) and adults (18–40 years). Inclusion of patients will be performed between May 2013 and February 2015 in the three steps described hereunder (Figure 1).

#### Patient pre-selection

British patients aged 6–40 years with at least one LABA and/or IC prescription during the past 12 months will be pre-selected from The Health Improvement Network, which is a collection of pseudo-anonymised electronic primary care medical records^7^ collected in approximately 550 general practices in the UK, with 3.6 million active patients.

In France, more than 700 general practitioners will perform a preliminary selection of asthma patients aged 6–40 years, with at least two prescriptions of LABAs and/or ICs during the past 12 months, regardless of associations with other controllers. General practitioners will record all asthma-related prescriptions during the past 12 months. A similar pre-selection will be conducted in community pharmacies: pharmacists will record all asthma-related prescriptions available during this time interval for patients aged 6–40 years, with at least one dispensing of LABAs and/or ICs and two pharmacy visits during these past 12 months.

#### Patient eligibility

From the pre-selection database, the research team will select, in both countries, patients on the basis of an additional inclusion criterion, i.e., ⩾6 months of prescribed coverage of one of the following therapy patterns during the past 12 months: ICs without LABAs, LABAs without ICs, LABAs and ICs as separate inhalers (LABAs+ICs) or fixed-dose combinations (figures available in the Supplementary Information). No change of therapy pattern will be allowed during the last 12 months.

The following exclusion criteria will be checked in pre-selection databases (for the UK) or during enrolment visit by a general practitioner or a pharmacist (for France): chronic oral corticosteroid use (⩾15 consecutive days during the past 3 months), history of omalizumab therapy and/or any other concomitant respiratory disease (chronic obstructive pulmonary disease, cystic fibrosis, pulmonary fibrosis, bronchiectasis and tuberculosis). In case of SAEx within 2 months before inclusion, patients will be re-contacted 4 months later, so that they will be free of recent SAEx when entering the cohort.

#### Patients’ enrolment

In France, general practitioners and pharmacists will invite eligible patients to participate in the study during a general practitioner or pharmacy visit. In the UK, practices will forward postal invitation packs to eligible patients, who will be invited to contact the logistic centre by phone or online. A consent acknowledgement will be collected before any data collection.

### Data collection

**Figure 2:**
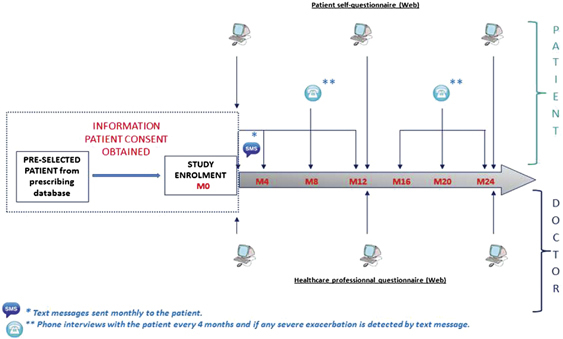
Summary of patient- and physician-reported data at inclusion and during follow-up.

Data collection schedule for patient-, caregiver- and health care professionals (HCP)-reported data is summarised in Figure 2.

#### Patient-reported data

##### Computerised-assisted telephone interviews and text messages

Trained interviewers will administer computerised-assisted telephone interviews (CATIs) to patients aged 12–40 years (parents/caregivers of patients aged 6–11 years) immediately after inclusion and every 4 months, to assess asthma control, asthma medication used during the past 4 months and SAEx occurrence. If a SAEx is reported, the asthma control- and medication-related questions will be repeated for the period before the SAEx, followed by additional questions (triggers, management). Patients will also receive monthly text messages inquiring about potential new SAEx; a positive answer will be followed by an additional CATI including the above-mentioned SAEx-related questions.

##### Online surveys

Patients and/or parents/caregivers will be requested to complete online surveys (adapted to age-specific requirements) at 12-month intervals on determinants of medication adherence, self-monitoring of symptoms, triggers and exacerbations management, quality of inhaler technique, quality of life, demographic and other background characteristics.

#### HCP-reported data

HCPs will complete online surveys on their routine asthma care and determinants of adherence support.

#### Electronic medical records/claims data

The Health Improvement Network data will be available in the UK, whereas refill and hospitalisation data will be obtained from the National Health Insurance System (SNIIRAM) in France.

### Measures

We present the main characteristics of the measures relevant to the primary research question regarding the safety of LABAs.

#### Outcomes

The primary outcome will be the occurrence of SAEx,^8–10^ operationalised as occurrence of patient-reported courses of oral corticosteroids (⩾3- day duration), unscheduled asthma-related medical contacts, emergency room visits, hospital admissions and death.

The secondary outcomes will be asthma control, measured with validated questionnaires—symptoms only Asthma Control Questionnaire (ACQ-5)^11^ and the Royal College of Physicians three questions,^12,13^ as well as the five-level European Quality of Life—5 Dimensions (http://www.euroqol.org/about-eq-5d/publications.html (25 November 2013)). For the latter, French and UK valuation set will be applied to estimate utilities in French and English participants, respectively.

#### Medication use and adherence

Medication use will be assessed via CATIs for each daily inhaled medication separately, with questions referring to different time intervals and behaviours: number of doses used the day before the interview, number of days with 0% and 100% adherence during the previous 7 days, number of days of non-use during the previous 4 weeks, treatment interruptions longer than a week in the previous 4 months, and medication overuse (for a specific time interval or occasional) in the previous 4 months. Patient/caregiver-reported drug exposure and adherence will be computed via algorithms developed in preliminary analyses.

### Therapy patterns, sample size and planned analyses

Studied exposure groups will be based on four initial therapy patterns. Preliminary results on pre-selected populations revealed that, for instance, only 2.4% of patients were prescribed LABAs without ICs in France, and virtually none in the UK. Given these low frequencies, patients under LABA monotherapy would be merged with those receiving ICs and LABAs as separate canisters, after having preliminarily verified their comparability. Hence, three groups have been considered for sample size power computation: LABAs with ICs in separate canisters or in monotherapy, LABA/ICs fixed-dose combinations and ICs without LABAs.

#### Sample size

Sample size computation for required power was based on differences in binomial proportions of SAEx between the reference ICs without LABAs group and LABAs with ICs in separate canisters or in monotherapy group. The hypothesis for the outcome frequency (24%) was based on asthma-related oral corticosteroid courses reported by patients during a 12-month period in a pharmacy-based study conducted in 2007 in ICs-treated patients.^14^ This hypothesis is conservative, as it may underestimate the true frequency of SAEx, which not only consider oral corticosteroid courses but also hospitalisations, unplanned medical contacts and death. Sample size calculations were based on the expected 1.3-fold higher frequency SAEx between the ICs without LABAs group and the other two groups. Considering a bilateral approach and balanced counts between groups, it was calculated that a total of 2,200 patients would be required given a statistical power of 80%, at a significance level of 5%, with a potential 20% loss to follow-up.

#### Planned analyses

##### Safety analyses

*Between-group comparisons*. As first approach, the time to the occurrence of the first SAEx will be compared between the initial exposure groups (ICs without LABAs group as reference) with survival analyses (Kaplan–Meier, Cox Model). The total number of SAEx per patient during a 12-month period will be also compared using Poisson regression.

*Cohort analyses with time-dependent variables*. Patients’ actual exposure to LABAs and ICs may change over follow-up owing to the prescriber or patient. In these analyses, such changes will be taken into account. Time-dependent variables, reflecting LABAs or ICs exposure over follow-up, will be constructed. The association between exposure to LABAs over time and the occurrence of SAEx will be investigated, after adjusting in particular for concomitant exposure to ICs. Survival analyses and hierarchical longitudinal models will be applied. Different markers of drug exposure and adherence will be successively explored.

*Case–crossover study and nested case–control study approaches*. As in a case–crossover design patients are their own controls, LABAs and ICs studied drug exposure patterns occurring just before a SAEx (case period) will be compared with those observed during a preceding regular CATI with no SAEx reported (control period), thus eliminating any potential influence of patients fixed characteristics. A nested case–control study approach will be also considered.

*Adjustment for asthma baseline severity*. Analyses will be adjusted for the different markers of asthma baseline severity, as it is a potential confounding factor when assessing LABA-related risk, except for the case–crossover approach.

Complementary analyses will be considered with the French patients, using claims data.

##### Adherence analyses

The relationships between medication adherence determinants and behaviours and asthma-related outcomes will be investigated based on a theoretical model of asthma management. These analyses will further examine the hypothesis that LABA risk in asthma may be partly owing to suboptimal adherence to ICs, with initial symptoms masked by concomitant LABA use, leading to severe and sudden SAEx. Moreover, they also aim to identify important and changeable causes of nonadherence from a patient, caregiver and HCP perspective, with a view to improving adherence support in primary care.

### Ethics

ASTRO-LAB study has been approved by Ethics and Regulatory Boards in both countries.

## Discussion

ASTRO-LAB presents several innovative aspects that will allow a unique perspective on LABA safety and asthma management, in real-life conditions. Data will be collected from complementary sources to assess patients’ drug exposure. This will allow assessing more elaborated markers of drug exposure and adherence to check the robustness of our findings.

The direct assessment of patients’ adherence to therapy, including potential changes over time, will enable us to distinguish the confounding role of inadequate adherence to ICs from LABA-specific risk, as differential adherence between LABAs and ICs may contribute to the occurrence of SAEx for patients receiving both classes in separate canisters.

Methodological limitations and practical difficulties must be acknowledged. The scarcity of patients prescribed LABAs in monotherapy may prevent any reliable conclusion for this non-recommended therapy pattern.^6,15^ Pre-selection process between countries will differ: pre-existing prescribing database in the UK versus an *ad hoc* prescription register collected by physicians themselves purposely for the study. This difference is owing to practical access to existing prescribing data (possible in the UK only, in the context of ASTRO-LAB). Nevertheless, the same inclusion/exclusion criteria will be eventually applied to all patients in both countries. A potential bias inherent to prospective studies will be that patients’ interaction with field study procedures (for example, questionnaires and CATIs) may modify their behaviours and beliefs regarding medication intake. Nonetheless, this potential bias will influence all treatment groups equally.

Practical difficulties have to be addressed in this multifaceted project. Different national regulatory requirements and health care systems between France and the United Kingdom compelled us to consider specific recruitment processes for each country, while attempting to maintain the two processes as similar as possible to minimise bias.

Given its unique perspective on asthma care, ASTRO-LAB will provide new information on LABA safety of substantial interest to regulators, HCPs, patients and the scientific community. Moreover, developing new methods of assessing drug exposure and adherence will make a valuable contribution beyond the field of asthma care. The investigation of multifaceted insight into asthma management in two different medical systems may be informative for the improvement of asthma care.

Further information can be found in the Supplementary Information.

## Notes

During the past 5 years, EVG has received funds for research, participations to congresses and consulting from ALK-ABELLO, BIF, MSD, ASTRA-ZENECA and CHIESI. During the past 5 years, EVG has been the main investigator of epidemiologic studies sponsored by GSK, MSD, CHIESI and PFIZER. The other authors declare no conflict of interest.
