# Supplementary material for: Assessment of the safety of long-acting β2-agonists in routine asthma care: the ASTRO-LAB protocol
Source: NPJ Prim Care Respir Med. 2015 Jun 18;25:15040–. doi: 10.1038/npjpcrm.2015.40 (PMC4498225; doi:10.1038/npjpcrm.2015.40)
Supplement: Supplementary Information [file npjpcrm201540-s1.doc]

**Supplementary information**

**Supplemental figure 1: Studied drug exposure patterns**

**
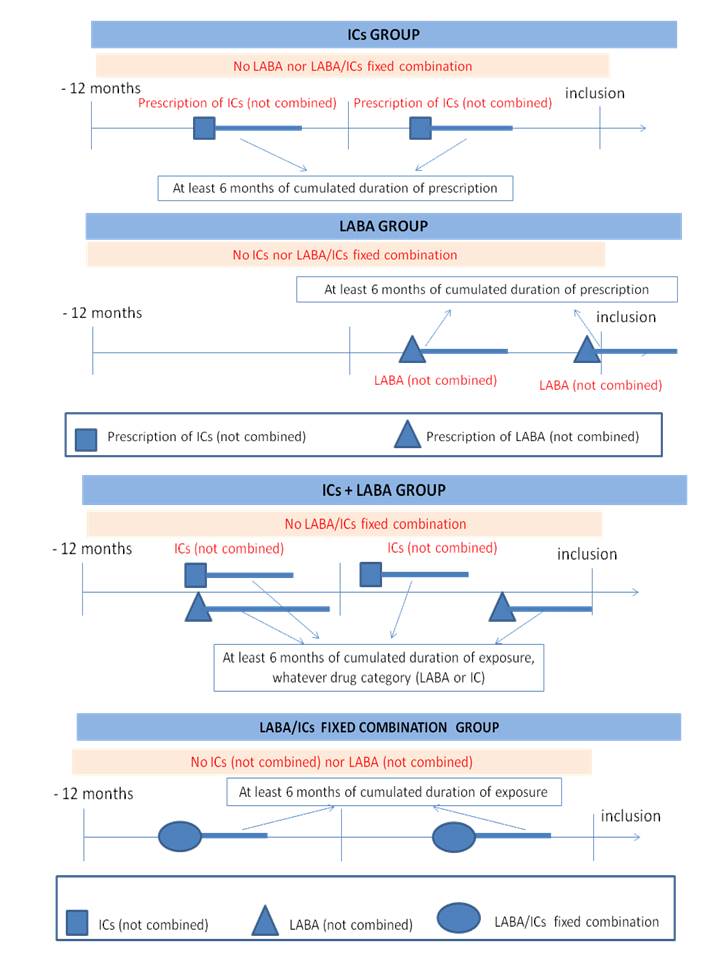
**

**Supplemental table 1:** Data collection schedule

|  | Month 0 | Every month | Every 4 months/ after SAEx | Month 12 | Month 24 |
| --- | --- | --- | --- | --- | --- |
| Data collected for (and from) HCPs |  |  |  |  |  |
| HCP contact details (France only) | X |  |  |  |  |
| HCP characteristics | X |  |  |  |  |
| HCP-reported content of asthma care (online) | X |  |  | X  (France only) | X  (France only) |
| HCP-reported determinants of adherence support (online) | X |  |  | X  (France only) | X  (France only) |
| Data collected for patients |  |  |  |  |  |
| From GPs (France only) |  |  |  |  |  |
| Patient medical characteristics | X |  |  | X | X |
| Diagnosis according to GP | X |  |  |  |  |
| Prescription of asthma drugs | X |  |  | X | X |
| Comorbidities and risk factors | X |  |  | X | X |
| Quality of inhaler technique |  |  |  |  | X  (France only) |
| From patient and/or caregivers (online) |  |  |  |  |  |
| Consent (assent 6-11 years old) | X |  |  |  |  |
| Determinants of asthma medication adherence | X |  |  | X | X  (France only) |
| Self-monitoring of symptoms | X |  |  | X | X  (France only) |
| Management of triggers | X |  |  | X | X  (France only) |
| Management of exacerbations | X |  |  | X | X  (France only) |
| Quality of inhaler technique | X |  |  | X | X  (France only) |
| Quality of life | X |  |  | X | X  (France only) |
| Demographics and other background characteristics | X |  |  | X | X  (France only) |
| From patients - telephone interview (CATI***) |  |  |  |  |  |
| Verbal consent (parents and teenagers/adults UK) | X |  |  |  |  |
| Exacerbation occurrence : oral corticosteroid course, hospital contacts, medical visits | X |  | X |  |  |
| Asthma Control | X |  | X |  |  |
| Adherence to asthma medication | X |  | X |  |  |
| Perception of SAEx causes (triggers) | X |  | X |  |  |
| Refill | X |  | X |  |  |
| From patients (text messages) |  |  |  |  |  |
| Exacerbation occurrence |  | X |  |  |  |
| From database |  |  |  |  |  |
| Drug dispensing and refills | X  (France only) |  |  |  | X  (France only) |
| Hospitalisation (claims for France / THIN for UK) | X |  |  | X | X  (France only) |
| GP / specialist visits (claims for France / THIN for UK) | X |  |  | X | X  (France only) |
| Patient characteristics, comorbidities (THIN for UK) | X |  |  | X |  |
| Practice characteristics (THIN for UK) | X |  |  | X |  |

**CATI: every 4 months on a regular basis + every time a SAEx was identified by text messages*

Abbreviations: HCP = health care provider; GP= general practitioner; CATI = computer-assisted telephone
